# What kind of articles does the JACMP seek to publish?

**DOI:** 10.1002/acm2.12069

**Published:** 2017-03-16

**Authors:** Michael D. Mills

With the transition of AAPM's publishing partner from Multimed to Wiley, the JACMP is reaching a wider and more diverse audience. It seems right once again to think about the types of articles that the JACMP wishes to publish. This Editorial therefore cuts two ways; those articles we definitely are seeking to publish, and those that we do not wish to publish and may decline without peer‐review.

Let me first elucidate some core philosophies of the JACMP:

- The Journal of Applied Clinical Medical Physics (JACMP) is an applied journal, which publishes papers that will help clinical medical physicists perform their responsibilities more effectively and efficiently for the increased benefit of the patient.
- The overriding focus of any JACMP paper should be its clinical application and value.
- If the treatment quality is better because of the paper or if the work to treat the patient is made more efficient, this is the type of paper we want.
- The JACMP does not want articles where the primary focus of the article is a contribution to the science of medical physics and the clinical component is missing or diminished.

These topical areas illuminate the types of manuscripts the JACMP Editorial Board would like to publish:

- CLINICAL Review Articles
- CLINICAL Radiation Oncology Physics
- CLINICAL Imaging Physics
- CLINICAL Nuclear Medicine Physics
- CLINICAL Radiation Measurements
- CLINICAL Non‐Ionizing Topics
- Radiation Protection & Regulations in Medicine
- Management of Technology in Clinical Medical Physics
- Professional Issues
- Education in Technology

These areas are representative of the types of manuscripts the JACMP Editors do not wish to publish:

- SCIENTIFIC Review Articles
- SCIENTIFIC Radiation Oncology Physics without a direct clinical application
- SCIENTIFIC Imaging Physics without a direct clinical application
- SCIENTIFIC Nuclear Medicine Physics without a direct clinical application
- SCIENTIFIC Radiation Measurements
- SCIENTIFIC Non‐Ionizing Topics
- Clinical Radiation Biology
- Clinical Biophysics
- Clinical Radiation Oncology
- Clinical Radiology

Yes, there are articles that contain both a clinical and a scientific emphasis, just as there are articles that touch on the clinical areas immediately above, while nevertheless, are primarily focused on topics within the JACMP publication space. If the article is hypothesis‐driven and is mostly about testing a scientific question, this article is likely more appropriate for the Medical Physics Journal. There is communication between the two AAPM academic journals, and occasionally an article submitted to one may be declined and recommended for the other. Additionally, the JACMP infrequently receives clinical articles that clearly belong in a clinical physician's journal, a radiobiology journal or a biophysics journal. I ask our authors to understand we are circumscribed by the publication space historically occupied by the JACMP and that if we recommend the article for another publication it is not a negative reflection on the article.

In short, if the paper does not help the practicing clinical medical physicist save time or do a better job for the patient, we would recommend submission to another journal. Thank you for your support of the JACMP and for your understanding of its publication goals.

## Conflict of Interest

None.

